# β-alanine supplementation improves tactical performance but not cognitive function in combat soldiers

**DOI:** 10.1186/1550-2783-11-15

**Published:** 2014-04-10

**Authors:** Jay R Hoffman, Geva Landau, Jeffrey R Stout, Matan Dabora, Daniel S Moran, Nurit Sharvit, Mattan W Hoffman, Yuval Ben Moshe, William P McCormack, Gil Hirschhorn, Ishay Ostfeld

**Affiliations:** 1 Institute of Exercise Physiology and Wellness, Sport and Exercise Science, University of Central Florida, Orlando, FL, USA; 2 Israel Defense Force, Medical Corps, Tel Hashomer, Israel; 3 School of Health Science, Ariel University, Center of Samaria, Ariel, Israel; 4 Israel Defense Forces, Combat Fitness Branch, Netanya, Israel

**Keywords:** Military performance, Marksmanship, Power, Physical performance, Supplements

## Abstract

**Background:**

There are no known studies that have examined β-alanine supplementation in military personnel. Considering the physiological and potential neurological effects that have been reported during sustained military operations, it appears that β-alanine supplementation may have a potential benefit in maintaining physical and cognitive performance during high-intensity military activity under stressful conditions. The purpose of this study was to examine the effect of 28 days of β-alanine ingestion in military personnel while fatigued on physical and cognitive performance.

**Methods:**

Twenty soldiers (20.1 ± 0.9 years) from an elite combat unit were randomly assigned to either a β-alanine (BA) or placebo (PL) group. Soldiers were involved in advanced military training, including combat skill development, navigational training, self-defense/hand-to-hand combat and conditioning. All participants performed a 4-km run, 5-countermovement jumps using a linear position transducer, 120-m sprint, a 10-shot shooting protocol with assault rifle, including overcoming a misfire, and a 2-min serial subtraction test to assess cognitive function before (Pre) and after (Post) 28 days of supplementation.

**Results:**

The training routine resulted in significant increases in 4-km run time for both groups, but no between group differences were seen (p = 0.597). Peak jump power at Post was greater for BA than PL (p = 0.034), while mean jump power for BA at Post was 10.2% greater (p = 0.139) than PL. BA had a significantly greater (p = 0.012) number of shots on target at Post (8.2 ± 1.0) than PL (6.5 ± 2.1), and their target engagement speed at Post was also significantly faster (p = 0.039). No difference in serial subtraction performance was seen between the groups (p = 0.844).

**Conclusion:**

Results of this study indicate that 4-weeks of β-alanine ingestion in young, healthy soldiers did not impact cognitive performance, but did enhance power performance, marksmanship and target engagement speed from pre-ingestion levels.

## Background

Investigations examining β-alanine ingestion in both recreational and competitive athletic populations have been consistent in demonstrating significantly greater performance during high-intensity physical activity than when these athletes are consuming a placebo [1-7]. The efficacy of β-alanine ingestion appears centered on its ability to enhance the quality of a workout by delaying skeletal muscle fatigue. The ergogenic properties of β-alanine by itself appear to be very limited. However, when β-alanine is absorbed into skeletal muscle it combines with histidine to form carnosine. It is carnosine which appears to provide the ergogenic benefit [8]. The primary role of carnosine is the maintenance of acid–base homeostasis through enhanced intra-muscular hydrogen ion (H^+^) buffering capacity [9]. Increasing intra-muscular carnosine concentration through β-alanine supplementation has been demonstrated to have ergogenic potential for maximal exercise lasting 60 sec - 240 sec [10]. Because carnosine is located in other excitable tissues other than skeletal muscle (such as the brain and heart), it may also have additional physiological roles [11-13].

Carnosine’s biological role as an antioxidant, antiglycating and ion-chelating agent suggests that it may have a potential role during oxidative stress, serving as a neuroprotector [11-13]. However, only one study has examined the effect of β-alanine ingestion on changes in carnosine concentrations in the brain [14]. Daily ingestion of 22.5 mmol·kg^−1^ of β-alanine in mice under stressful conditions resulted in an increase in carnosine concentrations in the cerebral cortex and hypothalamus, and an increase in brain derived neurotrophic factor in the hippocampus. In addition a decrease in 5-hydroxyindoleacetic acid concentrations, a metabolite of serotonin, was seen in the hippocampus. These changes, which also included improved time in a maze that contained anxiolytic compounds, resulted in the authors suggesting that β-alanine ingestion may have possible anxiolytic-like effects [14]. Although this has not been examined in a human model, it does provide an interesting basis for study. If β-alanine ingestion can increase brain carnosine concentrations in humans, it may provide a benefit in maintaining focus, alertness and cognitive function during highly fatiguing, high intense activity.

During prolonged, high-intensity military training or simulated combat exercises, significant decreases in physical and cognitive performance measures are often reported [15-18]. To compensate for the physiological and psychological fatigue associated with military training and combat, a number of pharmacological interventions have been examined. However, a recent commentary among the Medical Corps of the United States military has expressed a need to examine non-pharmacological alternatives to counteract the fatigue associated with military combat [19]. The use of dietary supplements among military personnel appears to be quite common. A recent study indicated that up to 72% of the Marines deployed to Afghanistan used a dietary supplement [20], while 53% of the soldiers at various military installations around the world (outside of the combat theater) indicated that they used dietary supplements on a regular basis [21]. However, little is known regarding the efficacy of many of these supplements as they relate to specific military performance.

To date, there are no known studies that have examined β-alanine supplementation in military personnel. Considering the physiological and potential neurological effects, it appears that β-alanine supplementation could have a potential benefit in preparation for prolonged, high intense military activity that requires maintaining high levels of physical performance, focus, and decision making ability under stressful conditions. The purpose of this study was to examine the effect of 28 days of β-alanine ingestion in military personnel while fatigued on physical and cognitive performance.

## Methods

### Subjects

Twenty male soldiers from an elite combat unit of the Israel Defense Forces (IDF) volunteered to participate in this double-blind study. Following an explanation of all procedures, risks and benefits, each participant provided his informed consent to participate in the study. The Helsinki Committee of the IDF Medical Corp approved this research study. Subjects were not permitted to use any additional dietary supplementation and did not consume any androgens or any other performance enhancing drugs. Screening for performance enhancing drug use and additional supplementation was accomplished via a health questionnaire completed during participant recruitment. Participants were from the same unit, but were from three different squads. Volunteers from each squad were randomly assigned to one of two groups. The randomization procedure involved that each volunteer from the same squad to be alternatively assigned to each group. Two participants dropped from the study, one participant fractured his leg during training, while the other participant no longer wished to participate. Each participant was from a separate group. Thus, a total of 18 participants were used in the final analysis. Using the procedures described by Gravettier and Wallnau [22] for estimating samples sizes for repeated measures designs, a minimum sample size of n = 8 was required for each group to reach a statistical power (1-β) of 0.80 based on the jump power changes reported by Hoffman et al. [4] The first group; (BA; age 20.1 ± 0.7 years; height: 1.79 ± 0.07 m; body mass: 78.3 ± 9.7 kg) consumed 6.0 g of β-alanine per day, while the second group (PL; age 20.2 ± 1.1 years; height: 1.80 ± 0.05; body mass: 79.6 ± 7.8 kg) consumed 6 g of placebo (rice flour). During the 4-week study period all participants from all squads participated in the same advanced military training tasks that included combat skill development, physical work under pressure, navigational training, self-defense/hand-to-hand combat and conditioning.

### Testing protocol

This randomized, double-blind, placebo controlled investigation was conducted at the unit’s training facilities, under the unit’s regular training protocols and safety regulations. Data collection occurred before (Pre) and after (Post) 28 days of supplementation. To create an acute fatigued state, each session required all participants to perform a 4 km run dressed in shorts, T-shirt and running shoes. Immediately following the 4 km run participants performed five countermovement jumps (CMJ). Participants then proceeded to put on their operational gear and weapon (12 kg) and ran a 120 m sprint. Following the sprint, participants proceeded as quickly as possible onto the shooting range and performed a 10-shot shooting protocol with their assault rifle. During the shooting, a planned misfire occurred that required the participant to correct and resume shooting. Immediately following the shooting drill all participants completed a serial subtraction test to assess cognitive function in a fatigued state.

### Performance measurements

#### Global positioning system

All participants were provided with an individual global positioning system (GPS) that they wore in a vest underneath their shirt. The GPS unit (MinimaxX, V4.3, Catapult Innovations, Victoria, Australia) was positioned in a posterior pocket on the vest situated between the participant’s right and left scapula in the upper-thoracic spine region. Information on velocity patterns was recorded during the 4 km run. Peak velocity, mean velocity, distance covered running at slow - moderate speed (< 4.44 m∙sec^−1^), distance covered running at high speed (4.44+ m∙sec^−1^), and the percent of total distance run at slow-moderate and high speeds were downloaded from the GPS receiver/transmitters. Data were collected at 10 Hz and all analysis was performed with the system software provided by the manufacturer. The validity and reliability of the GPS technology has been previously demonstrated [23].

#### Jump power

To quantify vertical jump power, participants performed five consecutive CMJ. During each CMJ participants stood with their hands on their waist at all times and were instructed to maximize the height of each jump, while minimizing the contact time with the ground between jumps. During each jump the participant wore a belt connected to a Tendo™ Power Output Unit (Tendo Sports Machines, Trencin, Slovak Republic). The Tendo™ unit consists of a linear position transducer attached to the end of the belt which measured linear displacement and time. Subsequently, the velocity of each jump was calculated and power determined. The average peak and mean power outputs for all five jumps were recorded. Intraclass correlations for the Tendo Unit and peak and mean vertical jump power in our laboratory has been R = 0.98, (SEM =106.2 W) and R =0.94 (SEM = 100.3 W), respectively.

#### Shooting performance

Targets were set at a 40-m distance from the firing line and were all headshots. Each shot that hit the target was considered accurate. Twenty targets were set up on the range. All participants were notified prior to the start of data collection which target they were required to shoot at. Immediately following the 120-m sprint, participants continued onto the shooting range and shot five times while kneeling and five times from a prone position with their assault rifle. Participants were instructed to shoot rapidly and accurately. While shooting each participant was required to handle a misfire in their weapon. The misfire was prearranged by the investigative team, which involved placing an empty bullet randomly into weapon’s magazine (weapon’s ammunition storage and feeding device). This required the participant to recognize and correct the misfire (clear the bullet) and continue to deliver fire at the designated target. The designed misfire was set to increase the stress of the shooting, with the participants already fatigued from the 4 km run, jumps and sprint with full gear. The number of accurate shots and the time required to perform these shots was recorded.

### Cognitive function

A modified version of the original Serial Sevens Test was employed to analyze cognitive function [24]. The test consisted of a two-minute timed written test in which participants were required to subtract the number 7 from a randomly generated four digit number, in order to measure how quickly and accurately they can compute a simple mathematical problem. The four digit number appeared on the top of the first column of a three column sheet of paper. Participants were provided the sheet of paper and asked to complete as many calculations as possible in the two-minute period. Participant and timer/scorer sat opposite each other during testing. The answers to the calculations were written underneath the initial number. Regardless of answer provided, participants were then required to subtract the number 7 from that new number. Participants were not told if their answer was correct or not. The number of correct answers was recorded. Intraclass correlations for this assessment has been determined in our laboratory to be R < 0.81 [25].

### Supplement schedule

The β-alanine supplement (CarnoSyn™) was obtained from Natural Alternatives International (San Marcos, CA, USA). Both the supplement and placebo were in tablet form and were similar in appearance. Participants in the supplement group were provided with 2 tablets of sustained-release β-alanine at a dose of (2 g per serving) three times per day (total β-alanine intake was 6 g per day) and subjects in the placebo group were provided with an equivalent amount of rice powder. Participants were instructed to consume the supplement following their meals with water. Each participant was provided with a bottle containing a week’s supply of tablets. All bottles were returned at the end of the week. All tablets left in the bottle were counted, recorded, and the next week’s bottle was provided to the participant. Supplementation occurred every day over a 28-day period.

### Statistical analysis

Data were analyzed using a 2 × 2 [treatment (BA, PL) × time (pretest, posttest)] mixed factorial ANOVA. Differences in the mean posttest performance values were determined by using analysis of covariance, with pretest values serving as the covariate. One-Way Analysis of Covariance (ANCOVA) was utilized to analyze differences between treatment groups. For effect size (ES), the partial eta squared statistic was reported and according to Green and colleagues [26] 0.01, 0.06, and 0.14 represents small, medium, and large effect sizes, respectively. An alpha level of p < 0.05 was used to determine statistical significance. Data were analyzed using SPSS v20 software (SPSS Inc., Chicago, IL).

## Results

Compliance for consuming the supplement or placebo was 97%. Four subjects within BA reported isolated incidences of paresthesia. Upon review, it was discovered that each of these soldiers combined 2 – 3 supplement doses for that day. No adverse events were reported in these participants or in any other participant consuming the supplement during the required time points. During the 4-week training period the decrease in body mass in BA (−1.3 ± 1.0 kg) was significantly greater (p = 0.014, ES = 0.34) than PL (−0.2 ± 0.6 kg).

Comparison of performance measures between BA and PL during the 4-km run is shown in Table 1. When collapsed across groups a significant increase (p = 0.019) in time for the 4-km run was observed from Pre to Post in both groups combined. However, no significant interactions were noted between the groups. Significant main effects for time were also noted for both peak (p = 0.045) and mean (p = 0.005) velocity (both variables decreased, meaning that the soldiers ran slower) during the 4-km run, and no significant interactions were observed between the groups in either velocity measure. The distance run at low to moderate velocities was significantly greater at Post than Pre (p = 0.010) for both groups combined, however no significant interactions were seen between the groups. The distance run at high velocity was significantly reduced for both BA and PL (p = 0.022), and no significant interaction was noted. The percent distance ran at low to moderate velocity was significantly increased (p = 0.021), while the percent distance ran at high-intensity was significantly lower, for both groups combined (p = 0.019). No between group differences were observed in either variable.

**Table 1:** Running velocities during 4-km run

**Variable**	**Group**	**Pre**	**Post**	**p value**	**ES**	**95% Confidence interval**
Peak velocity (m · sec^−1^)	BA	5.84 ± 0.63	5.46 ± 0.26	0.597	.02	5.16 – 5.71
	PL	5.69 ± 0.46	5.51 ± 0.50			5.26 – 5.80
Average velocity (m · sec^−1^)	BA	4.25 ± 0.22	4.13 ± 0.27	0.729	.01	3.96 – 4.24
	PL	4.18 ± 0.19	4.11 ± 0.19			3.99 – 4.28
Low – moderate running velocity (< 4.4 m · sec^−1^)	BA	2811 ± 605	2957 ± 672	0.224	.10	2571 – 3354
	PL	2827 ± 482	3297 ± 590			2900 - 3683
High running velocity (< 4.4 m · sec^−1^)	BA	1166 ± 610	1009 ± 675	0.364	.06	604 - 1399
	PL	1143 ± 485	748 ± 541			358 - 1153
% Distance run at low to moderate running velocity	BA	70.8 ± 16.2	74.3 ± 18.3	0.351	.06	64.4 – 84.6
	PL	71.3 ± 12.8	81.1 ± 14.4			70.9 – 91.0
% Distance run at high running velocity	BA	29.3 ± 16.1	25.4 ± 18.0	0.361	.06	15.4 – 35.2
	PL	28.8 ± 13.0	18.9 ± 14.4			9.1 – 29.0
4 K run time (sec)	BA	942.4 ± 39.3	962.6 ± 65.0	0.864	.002	929.4 – 1001.2
	PL	949.9 ± 46.2	963.9 ± 44.3			925.2 – 997.1

ES = Effect size.

**Figure 1:**
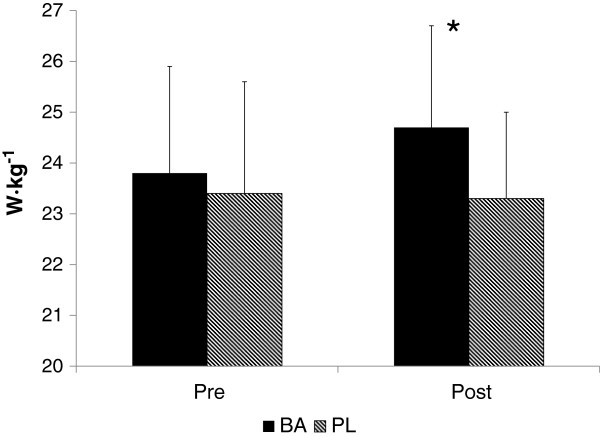
**Vertical jump relative peak power performance.** * = Significant difference between groups. W · kg^−1^ = Watts per kilogram body mass.

**Figure 2:**
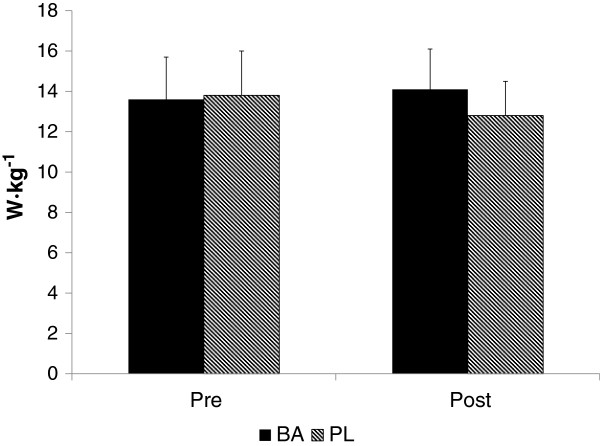
**Vertical jump relative mean power performance.** W · kg^−1^ = Watts per kilogram body mass.

Comparisons of vertical jump relative peak and mean power performances are shown in Figures 1 and 2, respectively. Relative peak power at Post was significantly greater for BA than PL (p = 0.034, ES = 0.27), while relative mean power for BA at Post (14.1 ± 1.7 w∙kg^−1^) was 10.2% greater (p = 0.139) than that observed for PL (12.8 ± 1.5 w∙kg^−1^). Although differences in mean jump power were not significantly different between the groups, the effect size of 0.14 is suggestive of a large effect due to the intervention (BA). No significant change in 120 m sprint velocity was seen from pre to post in either BA (4.65 ± 0.53 m · sec^−1^ and 4.45 ± 0.56 m · sec^−1^, respectively) or PL (4.49 ± 0.56 m · sec^−1^ and 4.35 ± 0.40 m · sec^−1^, respectively), and no differences between the groups were noted.

**Figure 3:**
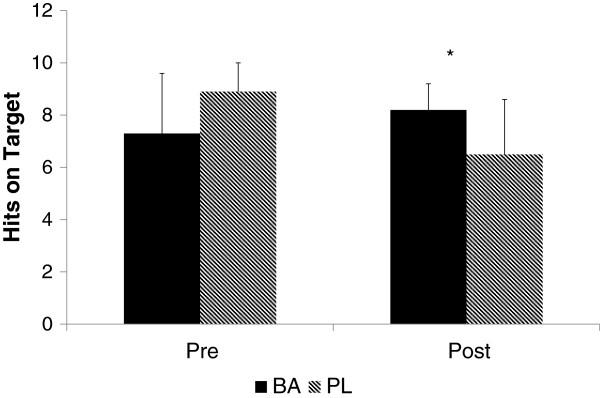
**Shooting accuracy reported as shots on target.** * = Significant difference between groups.

**Figure 4:**
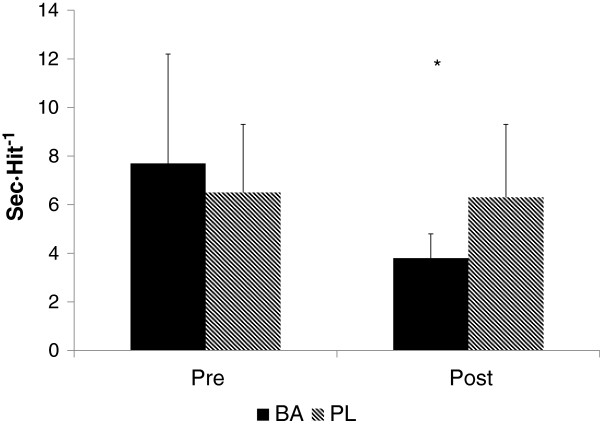
**Time per shot on target reported as seconds per accurate hit.** * = Significant difference between groups.

**Figure 5:**
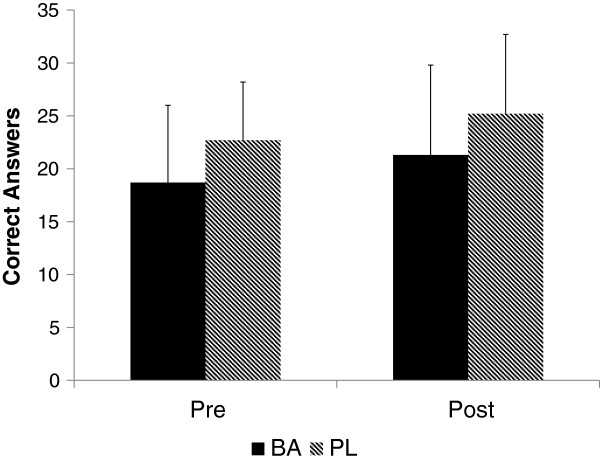
Serial subtraction test reported as number of correct responses.

The effect of the supplement on shooting accuracy and time per shot on target can be seen in Figures 3 and 4, respectively. A significantly greater (p = 0.012, ES = .38) number of shots on target was seen at Post for BA (8.2 ± 1.0) compared to PL (6.5 ± 2.1). The time per shot on target at Post was also significantly faster for BA than PL (p = 0.039, ES .27). When collapsed across groups, significant improvements in the serial subtraction test was seen from Pre to Post (p = 0.014), but no differences (see Figure 5) between the groups were seen (p = 0.844, ES = .003).

## Discussion

Results of this study demonstrate that 4 weeks of β-alanine supplementation during an intense military training period was effective in enhancing lower-body jump power and psychomotor performance (shooting accuracy) in soldiers of an elite IDF Combat unit, but did not appear to have any significant effects on cognitive function or running performance. While the benefits of β-alanine for athletic performance enhancement have been demonstrated in numerous studies [10,27,28], this investigation appears to be the first to provide evidence of β-alanine’s potential efficacy in military specific tasks.

During the 4 week study period all participants were participating in advanced military training that included combat skill development, physical work under pressure, navigational training, self-defense/hand-to-hand combat and conditioning. This training program, as expected, appeared to be quite fatiguing as significant performance decrements were seen in 4-km run performance for both groups. Previous research has shown that intense military training from one to eight weeks can result in significant decreases in strength and power [16,18]. In addition to the physical performance decrements associated with intense military training, decreases in shooting performance [29] and cognitive function [30] have also been reported. To defend against the physical and cognitive performance decrements related to intense and sustained military action, several studies have examined the efficacy of various stimulants and other pharmacological agents [15,31,32]. These studies have shown that such interventions can be very effective in sustaining military performance. For instance, Lieberman and colleagues [15] reported that caffeine ingestion following 72 hours of sleep deprivation was able to maintain cognitive function and mood, while Estrada et al., [31] noted that helicopter pilots provided 3 doses of modafinil (a vigilance promoting drug used to treat sleepiness) at 4-hour intervals during a 40-hour period of sustained wakefulness were able to maintain alertness, cognitive function and feelings of well-being. However, concerns have been raised regarding the safety and potential side effects associated with pharmaceutical agents, and calls for a greater effort in exploring non-pharmacological alternatives for military populations have been published [19]. Despite the popularity of dietary supplements in both deployed and garrisoned soldiers [20,21], little is known regarding the efficacy of many of these supplements as they relate to specific military performance. The results of the present study demonstrate the ergogenic benefits of β-alanine ingestion on enhancing tactical performance in elite combat solders.

Four weeks of β-alanine ingestion with dosages similar to the one used in the present study has been shown to elevate muscle carnosine concentrations by 60% [1]. Elevations in muscle carnosine has been demonstrated to enhance intracellular muscle buffering capacity and delay fatigue during high-intensity anaerobic exercise [9,10], but its benefits during endurance activity has proved to be inconclusive. During the 4-km run performed in this study we were unable to show any significant advantage related to β-alanine ingestion. There have only been a limited number of studies examining the effects of β-alanine ingestion and endurance performance. Jordan and colleagues [33] reported that following 4 weeks of β-alanine ingestion in participants who were not training aerobically during the supplement period a delay in blood lactate accumulation was seen, but a decrease in aerobic capacity was also noted. The physiological role of carnosine in muscle does not provide a strong mechanism for enhancing aerobic exercise performance. However, it may increase the time spent running at higher velocities. Although our results do not support this statistically, a 34.9% difference was seen between BA and PL in the distance run at a high velocity, which warrants further exploration with larger sample sizes. Regardless, the 4-km run performed in this investigation was primarily done to increase the fatigue of the soldiers prior to the shooting and cognitive function measures.

Following the 4-km run, subjects were required to perform a jump power test. The greater power performance observed in BA compared to PL was consistent with other studies demonstrating the fatigue resistant effects of β-alanine during high-intensity activity [34-36]. Derave and colleagues [34] reported that 4 weeks of β-alanine supplementation (4.8 g∙day^−1^) was able to delay fatigue during repeated bouts of isokinetic exercise and Van Thienen and colleagues [36] noted improved 30-sec sprint performance following a 110-min time trial. Each of those studies demonstrated a delay in fatigue following an acute exhaustive exercise protocol. Kern and Robinson [35] reported enhanced anaerobic exercise performance following a prolonged period (8-weeks) of high-intensity training in athletes supplementing with β-alanine compared to a placebo. The present study provides additional support of the benefits associated with 4-weeks of β-alanine supplementation in delaying fatigue.

Shooting performance has been shown to be sensitive to acute fatiguing activity [29,32]. Gillingham and colleagues [32] demonstrated that caffeine intake before and following exhaustive exercise (2.5-hr loaded march and 1.0-hr sandbar wall construction) improve target detection, marksmanship and engagement speed during simulated combat. This present study is the first to demonstrate that the fatigue resistant effects afforded by β-alanine ingestion can also improve marksmanship and target engagement speed following fatiguing exercise. Considering that this study did not measure muscle or brain carnosine concentrations, it is unclear if this played any role in the improvements observed or whether another mechanism associated with β-alanine ingestion may be responsible for the improvement in target acquisition and marksmanship.

Fatigue during sustained and highly intense combat situations may jeopardize rapid judgment in differentiating friend from foe. The subjects in the present study were required to overcome a misfire in their weapon, and then following their shooting performance complete mathematical problems while seated. The participants in BA were able to perform their 10 shots (30.2 ± 5.8 sec) faster than PL (37.7 ± 13.9 sec), but this 24.8% difference between the groups was not statistically different (p = 0.161). However, when the time was calculated relative to the number of shots on target, BA was significantly faster than PL. Whether this was related to an improved neurological benefit is not clear; however it is clear that β-alanine supplementation directly led to enhanced marksmanship and rate of target acquisition, suggestive of improved psychomotor performance. Furthermore, the misfire in the weapon was similar for all participants and similar in both Pre and Post assessment periods. It is possible that the familiarity with how to handle the misfire for both groups also contributed to the similar completion time for the 10 shots.

There were several limitations with this study. Considering that no previous studies examined the role of β-alanine on cognitive function, the statistical power analysis used to determine subject size was based upon previous studies examining physical performance. In regards to the cognitive aspects of this study, the statistical power may not have been appropriate. In addition, the benefits of performing a field study are often offset by the inability to control all aspects of the participant’s daily activity. For instance, the structure of the training did not provide an opportunity to control or record the participant’s diet. However, considering that participants were provided the same meals we made certain assumptions that the dietary intake would be similar between groups. The training schedule also forced several volunteers to miss their scheduled ingestion time for the supplement or placebo. It was in those situations where incidences of paresthesia occurred when the volunteer ingested multiple doses at the same time. Although volunteers were required to show the empty bottle and receive the following week’s supply at the end of each week, the daily control for ingestion during meals was not possible. However, this study provided a unique opportunity to examine the efficacy of this supplement under real-life conditions involving military operations. This opportunity is not common and the results provided important information for potential dietary interventions on sustaining tactical performance in stressful conditions.

## Conclusions

The results of this study did not provide any evidence in support of β-alanine’s role on enhancing cognitive function in fatigued soldiers. It is likely that the serial subtraction test performed with participants seated was not sufficient to ascertain the potential effects that β-alanine may have in improving cognitive performance following fatiguing activity. This study demonstrated that β-alanine ingestion for 4 weeks in young, healthy soldiers in an elite combat unit can enhance jump power performance, marksmanship and target engagement speed. These improvements occurred following 4 weeks of highly intense training and an acute fatiguing event (4-km run). The results of this study were unable to support any cognitive benefits from the 4-week supplement period. In consideration of the highly intense and fatiguing nature of sustained combat and prolonged military training, ingestion of β-alanine does appear to provide specific benefits for military personnel.

## Competing interests

All authors declare that they have no competing interests.

## Authors’ contributions

JRH, GL and IO were the primary investigators, supervised all study recruitment and data analysis. JRH, GL, MD, JRS, YBM, GH and IO assisted in the design of the study, JRH and JRS performed the statistical analysis, JRH supervised the manuscript preparation, JRS, JRH, DSM, and IO helped draft the manuscript. JRH, GL, DSM, NS, MWH, WPM and IO assisted with data collection and data analysis. All authors read and approved the final manuscript.
